# Sex-Dependent Effects of HO-1 Deletion from Adipocytes in Mice

**DOI:** 10.3390/ijms18030611

**Published:** 2017-03-11

**Authors:** Peter A. Hosick, Mary Frances Weeks, Michael W. Hankins, Kyle H. Moore, David E. Stec

**Affiliations:** 1 Department of Physiology & Biophysics, Center for Excellence in Cardiovascular-Renal Research, University of Mississippi Medical Center, 2500 North State St, Jackson, MS 39216, USA; hosickp@mail.montclair.edu (P.A.H.); mfweeks@crimson.ua.edu (M.F.W.); mhankins@umc.edu (M.W.H.); kmoore4@umc.edu (K.H.M.); 2 Department of Exercise Science and Physical Education, Montclair State University, Montclair, NJ 07043, USA

**Keywords:** obesity, diabetes, bilirubin, Cre recombinase, insulin resistance, adiponectin

## Abstract

Induction of heme oxygenase-1 (HO-1) has been demonstrated to decrease body weight and improve insulin sensitivity in several models of obesity in rodents. To further study the role of HO-1 in adipose tissue, we created an adipose-specific HO-1 knockout mouse model. Male and female mice were fed either a control or a high-fat diet for 30 weeks. Body weights were measured weekly and body composition, fasting blood glucose and insulin levels were determined every six weeks. Adipocyte-specific knockout of HO-1 had no significant effect on body weight in mice fed a high-fat diet but increased body weight in female mice fed a normal-fat diet. Although body weights were not different in females fed a high fat diet, loss of HO-1 in adipocytes resulted in significant alterations in body composition. Adipose-specific HO-1 knockout resulted in increased fasting hyperglycemia and insulinemia in female but not male mice on both diets. Adipose-specific knockout of HO-1 resulted in a significant loss of HO activity and a decrease in the protein levels of adiponectin in adipose tissue. These results demonstrate that loss of HO-1 in adipocytes has greater effects on body fat and fasting hyperglycemia in a sex-dependent fashion and that expression of HO-1 in adipose tissue may have a greater protective role in females as compared to males.

## 1. Introduction

Obesity continues to be a significant health concern in the United States and globally. Recent reports indicate that global obesity prevalence will reach 18% in men and surpass 21% in women by 2025 [1]. Obesity is commonly associated with other cardiovascular and metabolic disorders such as hypertension, diabetes, coronary heart disease and inflammation [2]. In addition, obesity is the leading cause of non-alcoholic fatty liver disease, which has increased from 46% to 75% of all chronic liver disease from 2005 to 2008 [3]. NAFLD also leads to hepatic insulin resistance that contributes to the development of type II diabetes, a major risk factor for cardiovascular disease [4]. Given the severity of the obesity epidemic and its complications, a greater understanding of the factors that promote and protect against the harmful effects of obesity is warranted.

Heme oxygenase (HO) is a member of the heat-shock family of proteins where it plays a critical role in the recycling of heme in the body [5]. HO enzymes consist of two isoforms, heme oxygenase-1 (HO-1) and heme oxygenase-2 (HO-2) and each breaks down heme to biliverdin, carbon monoxide, and free iron. Chemical induction of HO-1 has been demonstrated to prevent obesity, improve insulin sensitivity, and increase metabolism in numerous rodent models of obesity and diabetes [6,7,8,9,10,11,12]. Importantly, systemic inhibition of HO-1 activity has been demonstrated to block all of the beneficial actions of these various chemical inducers [8,9,10,11]. While chemical induction of HO-1 has been consistently demonstrated to have beneficial effects in obesity, adipose-specific overexpression of HO-1 has been reported to have no effect on the development of dietary-induced obesity and subsequent insulin resistance [13].

Chemical induction of HO-1 has been reported to activate adiponectin release from adipocytes and increase the circulating levels of adiponectin in the plasma [6,8,12,14]. HO is believed to increase adiponectin levels by acting as a molecular chaperone as well as directly increasing adiponectin levels in adipose tissue [15]. Adiponectin has many beneficial effects on metabolism and protects against the development of obesity-associated cardiovascular disease [16,17,18]. Another mechanism by which HO-1 may be protective against diabetes and obesity is through its antioxidant actions. HO increases cellular antioxidant capabilities through breakdown of heme and generation of CO and bilirubin and induction of ferritin [5]. Several studies have documented that elevated glucose levels observed in human and rodents with obesity decrease HO-1 expression [19,20].

The goal of this study was to determine the role of adipocyte HO-1 in the regulation of body weight and glucose metabolism under basal conditions and in response to dietary-induced obesity in male and female mice. In order to determine the role of HO-1 in adipose tissue, we created an adipose-specific knockout model of HO-1 using floxed HO-1 containing mice which were crossed with mice expressing the Cre recombinase expressed under the control of the adiponectin promoter [21,22].

## 2. Results

### 2.1. Adipocyte-Specific Knockout of HO-1 Has No Effect on Body Weight, but Alters Body Composition in Female Knockout Mice Fed a High-Fat Diet

**Figure 1:**
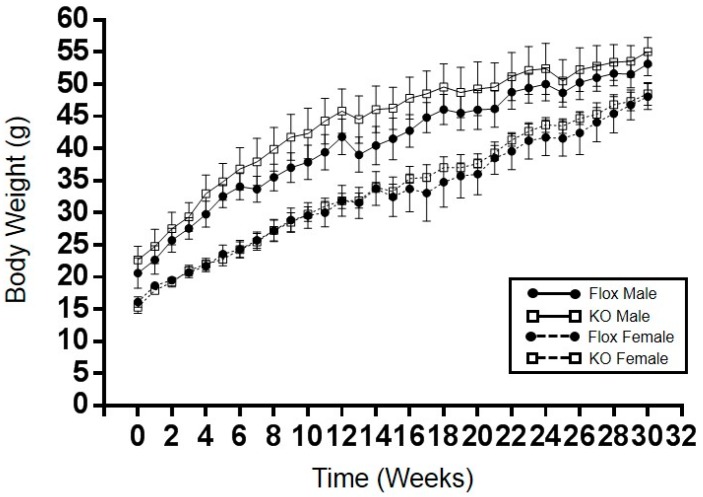
Weekly body weights of male and female Flox and KO (knockout) mice placed on a high-fat diet. No significant effect of on body weight was detected between males and females. Flox males, *n* = 8, KO males, *n* = 7, Flox and KO females, *n* = 6.

**Figure 2:**
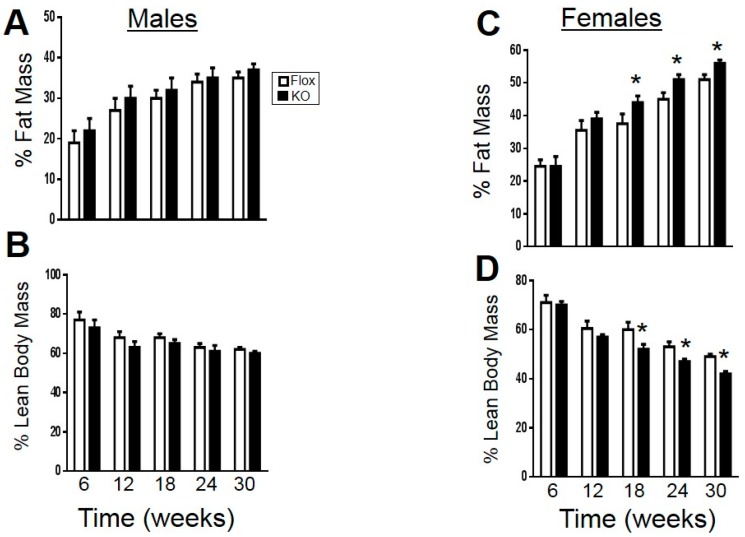
Fat and lean body mass in Flox and KO mice placed on a high-fat diet: (**A**) fat mass in males; (**B**) lean mass in males; (**C**) fat mass in females; and (**D**) lean mass in females. * *p* < 0.05 compared to corresponding value in Flox females. Flox males, *n* = 8, KO males, *n* = 7, WT and KO females, *n* = 6.

Adipocyte-specific knockout of HO-1 had no significant effect on high-fat diet induced obesity over the 30-week period in both male and female mice (Figure 1). Female adipocyte-specific knockout females did display a significantly higher body weight when maintained under a normal-fat diet as compared to Flox female mice (Table 1). Body composition in male mice fed a high-fat diet was not different between knockout and Flox mice (Figure 2A,B); however, female knockout mice did display increased fat mass and decreased lean mass over the last 12 weeks of the high-fat diet (Figure 2C,D).

### 2.2. Adipocyte-Specific HO-1 Knockout Results in Sustained Increases in Fasting Blood Glucose Levels and Hyperinsulinemia in Female but Not Male Mice

**Figure 3:**
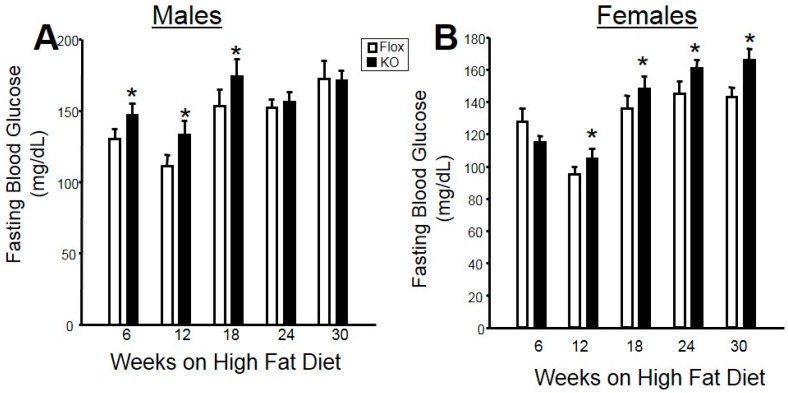
Fasting blood glucose levels in (**A**) male and (**B**) female mice over 30 weeks of high-fat diet. * *p* < 0.05 compared to the corresponding value in Flox mice.

**Figure 4:**
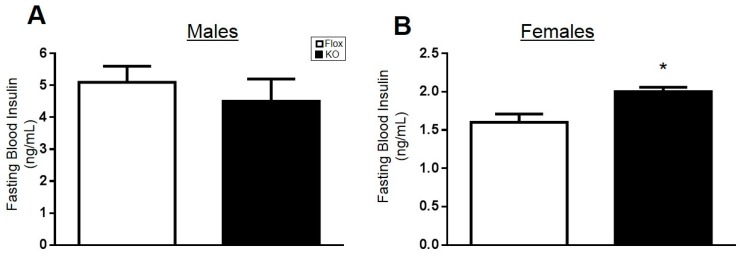
Fasting blood insulin levels in (**A**) male and (**B**) female mice at week 30 of a high-fat diet. * *p* < 0.05 compared to the corresponding value in Flox mice.

Fasting blood glucose levels were measured every six weeks in male and female mice on a high fat diet. In male mice, adipose-specific HO-1 knockout increased fasting blood glucose levels early in the study from Weeks 6 to 18; however, blood glucose levels were not different over the last 12 weeks of the study between the two genotypes (Figure 3A). Likewise, no differences in fasting blood insulin levels were observed between male Flox and knockout (KO) mice fed a high fat diet (Figure 4A). Fasting blood glucose and insulin levels were also not different between Flox and KO male mice fed a normal fat diet (Table 1). In contrast, significant differences in fasting blood glucose levels were observed between female Flox and KO mice fed a high fat diet from Week 12 to the end of the study (Figure 3B). Fasting blood insluin levels were also increased in female KO as compared to Flox mice fed a high fat diet (Figure 4B). Both fasting glucose and insulin levels were increased in the blood of KO versus Flox mice fed a normal fat diet as well (Table 1).

### 2.3. Adipocyte-Specific HO-1 Knockout Mice Exhibit Alterations in Adipose HO-1 Activity without Any Changes in Kidney or Liver HO-1 Activity and Express dsRed Protein Following Cre-Mediated Deletion of the HO-1 Allele

**Figure 5:**
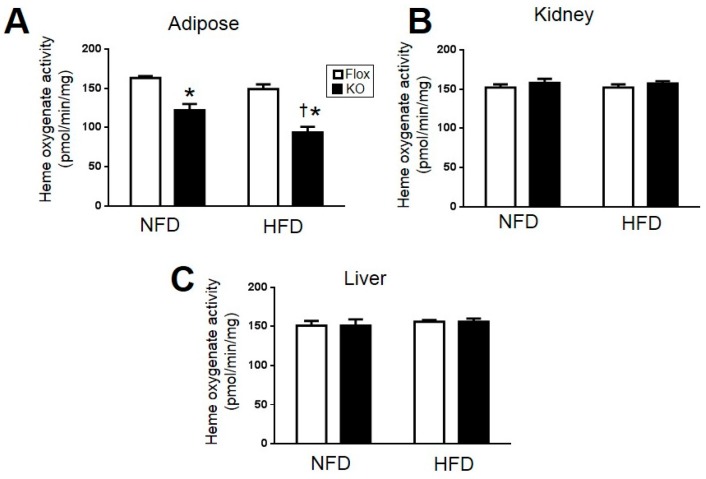
Heme oxygenase activity in the: (**A**) adipose tissues; (**B**) Kidney; and (**C**) Liver of Flox and KO male mice after 30 weeks of normal fat diet (NFD) and high-fat diet (HFD). * *p* < 0.05 compared to the corresponding value in Flox mice. † *p* < 0.05 compared to NFD. *n* = 6/group.

**Figure 6:**
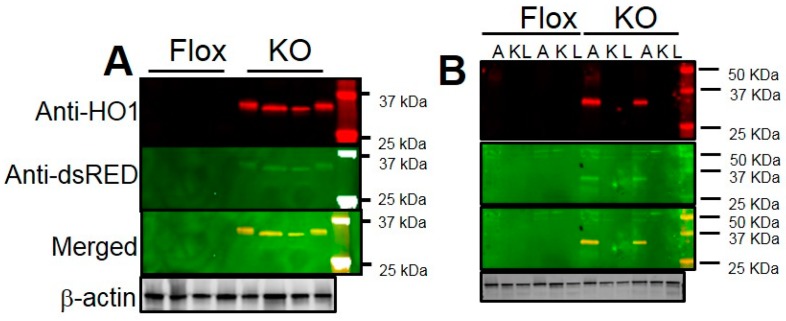
(**A**) Representative Western blot from adipose tissue of male Flox and KO mice after 30 weeks of high-fat diet (HFD); (**B**) Representative Western blot from of male Flox and KO mice adipose tissue (A), kidney (K), and liver (L) of Flox and KO mice after 30 weeks of high-fat diet with the anti-HO-1 antibody (**Red**) or anti-RFP (**dsRed**) antibody (**Green**) as well as merged image showing each antibody detects the same band in the adipose but not kidney and liver of KO mice. Levels of β-actin demonstrate equal loading of samples.

In order to determine the specificity of HO-1 deletion, HO-1 activity was measured in adipose, kidney, and liver of Flox and KO mice after normal and high-fat feeding. Adipose HO-1 activity decreased in KO mice as compared to Flox mice (Figure 5A). Interestingly, a significant decrease in HO-1 activity was observed between Flox mice fed a high-fat as compared to normal fat diet (Figure 5A). No differences in HO-1 activity between genotype and diet were observed in the kidney or liver demonstrating the specificity of the HO-1 knockout to adipose tissue (Figure 5B,C). Specificity of adipose-specific deletion of HO-1 was also confirmed by Western blot from adipose, kidney, and liver using antibodies to both HO-1 as well as sdRed. The HO-1 antibody detected a band ~35 kDa in size slightly larger than HO-1 (32 kDa) in adipose (Figure 6A,B) but not liver or kidney of adipose-specific HO-1 KO mice (Figure 6B). This band was also detected by the anti-dsRed antibody in adipose and not other tissues (Figure 6). A duel image clearly demonstrates that these antibodies detect the same protein in the adipose but not in the kidney or liver of adipose-specific HO-1 KO mice. We were not able to detect endogenous levels of HO-1 with the anti-HO-1 antibody in Flox mice which may be due to the fact that HO-1 has been previously reported to be down-regulated by HFD in the adipose tissue of mice [9].

### 2.4. Loss of HO-1 Decreases Adiponectin, PGC1α, and SIRT1 and Increases Markers of Inflammation in Adipose

**Figure 7:**
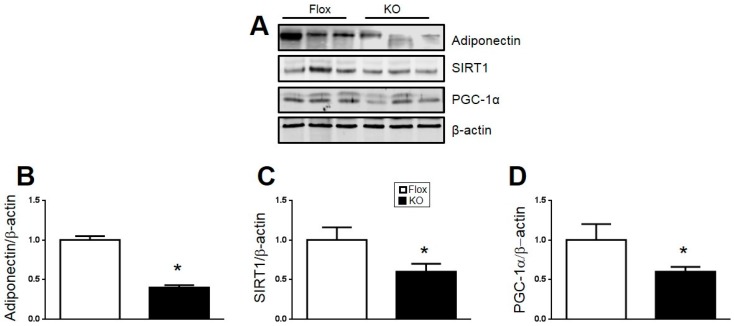
(**A**) Representative Western blots from adipose of male mice after 30 weeks of high-fat diet; (**B**) protein levels of Adiponectin; (**C**) protein levels of Sirtuin-1 (SIRT1); and (**D**) protein levels of peroxisome proliferator-activated receptor gamma coactivator 1-α (PGC-1α). * *p* < 0.05 compared to the corresponding value in Flox mice. *n* = 6/group.

**Figure 8:**
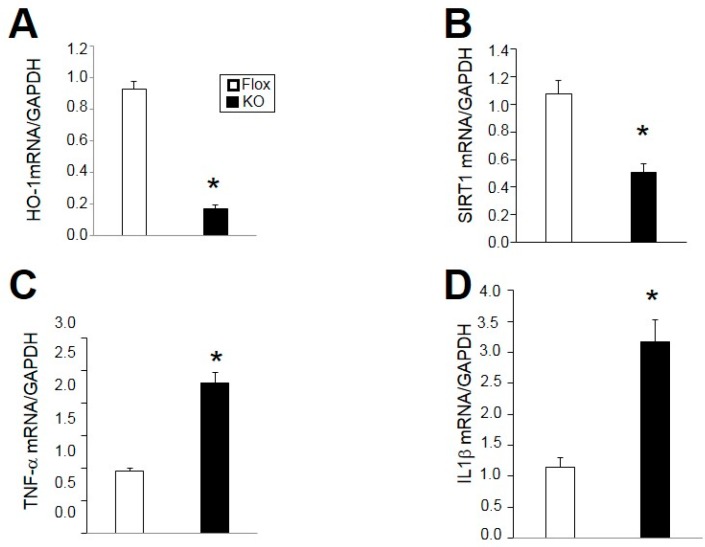
Gene expression levels of: (**A**) heme oxygenase-1 (HO-1); (**B**) Sirtuin-1 (SIRT1); (**C**) Tumor necrosis factor-α (TNF-α); and (**D**) Interleukin-1β (IL1β) in adipose of male mice after 30 weeks high fat diet. * *p* < 0.05 compared to the corresponding value in Flox mice. *n* = 4/group.

The levels of adiponectin, PGC1α, and SIRT1 were determined by Western blot of adipose tissue from Flox and KO mice. Levels of all of these proteins were significantly reduced in the adipose of KO compared to Flox mice (Figure 7). Next, we utilized real-time PCR to confirm the decrease in HO-1 in adipose of KO as compared to flox mice (Figure 8A). The decrease in SIRT1 protein was further confirmed by a similar decrease in SIRT1 mRNA in KO as compared to Flox mice (Figure 8B). An increase in two markers of inflammation, TNF-α and IL1β was also observed in the adipose of KO as compared to Flox mice (Figure 8C,D).

## 3. Discussion

Several studies have highlighted the beneficial action of chemical induction of HO-1 to lower body weight, normalize insulin resistance and improve the adipokine profile in rodent models of obesity [6,8,9,10]. However, the role of selective adipocyte HO-1 deficiency on the regulation of body weight and composition under basal conditions and in response to dietary-induced obesity was not previously known. The results of our study demonstrate the loss of adipocyte HO-1 has a greater effect on body weight and composition in female versus male mice. The lack of effect of adipocyte-specific deletion of HO-1 on metabolic phenotypes in male mice is similar to that observed in male mice in which HO-1 was specifically overexpressed in the adipose tissue [13]. However, the effect of adipose-specific overexpression on female mice with regard to body weight, composition, and insulin sensitivity was not reported in this study [13]. The role of sex differences in metabolic response to feast and famine has long been known [23]; however, only recently have we begun to think about sex differences in the development of obesity and responses to different intervention treatments [24,25]. Previous studies have demonstrated that although female mice were resistant to the weight lower actions of chemical induction of HO-1 with cobalt protoporphyrin (CoPP), they still exhibited beneficial actions on insulin resistance, blood pressure, and inflammation [9]. While this study did report the lack of effect of HO-1 induction on body weight, the effect on body composition was not determined [9]. The results of the present study demonstrate that loss of adipocyte HO-1 has significant effects on body composition to increase fat mass and lower lean mass independent of any changes in total body weight. These alterations in body composition in female adipocyte-specific HO-1 KO mice are associated with increased insulin resistance exhibited by increased fasting blood glucose and insulin levels in mice fed both normal and high-fat diets. These results suggest that adipose HO-1 may play a greater protective role to preserve insulin sensitivity in females versus males. Several studies have highlighted the important role that alterations in sex hormones play in the regulation of blood pressure in males and females [26,27]. It is possible that alterations in the levels of sex hormones following adipose-specific deletion of HO-1 contribute to the metabolic disturbances observed in female mice. This possibility needs to be further examined in future studies.

The role of sex hormones in the regulation of HO-1 has not been extensively studied. One study examining the sex influences of hepatic expression of HO1 following trauma and hemorrhage found HO-1 expression and activity were enhanced in females as compared to males [28]. Another study demonstrated that female streptozotocin-induced diabetic rats exhibited greater HO-1 induction as compared to male rats [29]. Likewise, female Wistar rats were found to exhibit elevated cardiac levels of HO activity and expression, which may play a role in the sexual dimorphism of cardiovascular ischemia-induced injury [30]. Lastly, HO-1 was found to be significantly elevated in the adipose tissue of women with polycystic ovary syndrome (PCOS) suggesting that the HO-1 system may be playing a beneficial compensatory role in the adipose tissue of these PCOS women [31].

The “HO-1/adiponectin axis” has been uncovered as a novel regulatory element for the beneficial actions of HO-1 induction in several models of obesity and cardiovascular disease [6,8,14,32,33]. HO-1 increases adiponectin levels in both adipose tissue as well as circulating levels in the plasma [6,11,12]. The mechanism by which HO-1 increases adiponectin levels is adipose tissue is unclear, and it could be species specific as HO-1 induction was reported to have no effect on adiponectin levels in cultured human adipocytes [34]. It is also possible that induction of HO-1 increases circulating adiponectin levels through release from non-adipose derived sources. The data from the current study support a role for HO-1 in the regulation of adiponectin levels in the fat as adipose-specific deletion of HO-1 resulted in decreased adiponectin protein levels in the fat. Another recently identified pathway by which HO-1 may be protective is through the Sirtuin1 (SIRT1) pathway. The SIRT1 pathway is a key regulator of metabolism and is an emerging target for the treatment of obesity [35,36]. The intersection of these two pathways has recently emerged in studies demonstrating the importance of SIRT1 in the protective actions of HO-1 induction in the liver in two different models of dietary-induced obesity with fatty liver disease [37,38]. The down-regulation of these pathways in the adipose tissue had no significant effect on the degree of obesity or insulin resistance in male mice; however, it is possible that alterations in these pathways contributed to the alterations in body weight, composition and insulin sensitivity in female mice. Future studies that alter these pathways either individually or together in female mice are needed to determine their roles definitively.

Obesity and inflammation have been extensively linked, but it is not clear if obesity drives the inflammatory state or increased inflammation drives obesity [2,16]. TNF-α and IL1β, two markers of inflammation, were increased in the adipose tissue of HO-1 KO mice. HO and its metabolites, bilirubin and carbon monoxide, possess potent anti-inflammatory actions [39,40,41,42]. Thus, loss of HO activity in adipocytes could set the stage for increased inflammation in these mice. However, this increase in the inflammatory state of the adipose tissue had little effect on body weight or composition in male mice but could contribute to the increase in fat mass and body weight as well as the insulin resistance observed in female mice. Given this possibility, it would be interesting to determine if sex-specific differences in anti-inflammatory treatment exist in adipose-specific HO-1 KO mice. However, these experiments are beyond the scope of the current study and would require additional studies in this model.

## 4. Materials and Methods

### 4.1. Animals

The experimental procedures and protocols of this study conform to the National Institutes of Health Guide for the Care and Use of Laboratory Animals and were approved by the Institutional Animal Care and Use Committee of the University of Mississippi Medical Center (protocol 1283, initial approval 2/2014, reapproved 1/2017).

Studies were performed on male and female Flox HO-1 and adipose-specific HO-1 knockout mice. Flox HO-1 mice are designed to delete exons 3–5 and activate red fluorescent protein (dsRed) upon cre-mediated deletion and maintained on a C57BL/6J genetic background as originally described [22]. Adiponectin-Cre mice were mice purchased from Jackson Labs (Bar Harbor, ME, USA) and were derived from the originally described colony and bred onto a C57BL/6J background [21]. Mice which contain both the Adiponectin-Cre and the flox HO-1 alleles are considered knockouts while mice which lack the Adiponectin-Cre and only contain the flox HO-1 allele are considered Flox mice. Mice were housed under standard conditions until 6 weeks of age after which time some mice were switched to a 60% high-fat diet (diet # D12492, Research Diets, Inc., New Brunswick, NJ, USA) other mice were allowed full access to standard laboratory chow. The groups of mice consumed each diet for 30 weeks.

### 4.2. Body Composition (EchoMRI)

Body composition changes were assessed at 6-week intervals throughout the study using magnetic resonance imaging (EchoMRI-900TM, Echo Medical System, Houston, TX, USA). MRI measurements were performed in conscious mice placed in a thin-walled plastic cylinder with a cylindrical plastic insert added to limit movement of the mice. Fat mass, lean mass, free water and total water were measured after brief exposure to a low-intensity electromagnetic field.

### 4.3. Fasting Glucose and Insulin

Following an eight hour fast a blood sample was obtained via orbital sinus under isoflurane anesthesia. Blood glucose was measured using an Accu-Chek Advantage glucometer (Roche, Mannheim, Germany). Fasting plasma insulin concentrations were determined by ELISAs (Rat/Mouse Insulin ELISA, Millipore, Temecula, CA, USA) as previously described [43].

### 4.4. Heme Oxygenase Assay

Heme oxygenase assays on lysates prepared from adipose, liver and kidney of male Flox and knockout mice were conducted at the end of the study as previously described [44,45,46]. Tissue was homogenized in 250 mM sucrose, 10 mM KPO_4_, 1 mM EDTA and 0.1 mM PMSF (pH 7.7) in the presence of protease inhibitors (2 µg/mL aprotinin, leupeptin and pepstatin). The homogenate was then centrifuged at 3000× *g* for 15 min at 4 °C and the supernatant collected. Protein concentration was measured using a Bio-rad protein assay with BSA standards. Reactions were carried out in a 1.2 mL containing: 2 mM glucose-6-phosphate, 0.2 unites glucose-6-phosphate dehydrogenase, 0.8 mM NADPH, 20 µM hemin, and 0.5 mg of lysates as previously described (2). The reactions were incubated for 1 h at 37 °C in the dark. The formed bilirubin was extracted with chloroform, and the change in optical density (ΔOD) at 464–530 nm was measured using an extinction coefficient of 40 mM/cm for bilirubin. HO activity was expressed as picomoles of bilirubin formed per hour per milligram of protein.

### 4.5. Quantitative Real-Time PCR Analysis

Total RNA was harvested from Flox and HO-1 knockout mice by lysing adipose tissue using a Qiagen Tissue Lyser LT (Qiagen, Germantown, MD, USA) and then extraction by 5-Prime PerfectPure RNA Tissue Kit (Thermo Fisher Scientific, Wilmington, DE, USA). Total RNA was read on a NanoDrop 2000 spectrophotometer (Thermo Fisher Scientific, Wilmington, DE, USA) and cDNA was synthesized using High Capacity cDNA Reverse Transcription Kit (Applied Biosystems, Thermo Fisher Scientific, Wilmington, DE, USA). PCR amplification of the cDNA was performed by quantitative real-time PCR using SYBR Green qPCR SuperMix (Applied Biosystems, Thermo Fisher Scientific, Wilmington, DE, USA). The thermocycling protocol consisted of 5 min at 95 °C, 40 cycles of 15 s at 95 °C, and 30 s at 60 °C and finished with a melting curve ranging from 60 to 95 °C to allow distinction of specific products. Normalization of samples occurred in separate reactions with primers to GAPDH mRNA.

### 4.6. Western Blot Analysis

Western blots were performed on lysates prepared from tissues collected at the end of the experiments. Tissue was homogenized in 250 mM sucrose, 10 mM KPO_4_, 1 mM EDTA and 0.1 mM PMSF (pH 7.7) in the presence of protease inhibitors (2 µg/mL aprotinin, leupeptin and pepstatin) as well as phosphatase inhibitors. Samples of 30 µg of protein were boiled in Laemmli sample buffer (Bio-Rad, Hercules, CA, USA) for 5 min and electrophoresed on 10 or 12.5% SDS-polyacrylamide gels and blotted onto nitrocellulose membrane. Membranes were blocked with Odyssey blocking buffer (LI-COR, Lincoln, NE, USA) for 2 h at room temperature and then incubated with primary antibodies overnight at 4 °C. Membranes were incubated with goat secondary antibodies anti-mouse (IR700) or anti-rabbbit (IR800) (LI-COR, Lincoln, NE, USA, 1:10,000) for 1 h at room temperature. Membranes were visualized using an Odyssey infrared imager (LI-COR, Lincoln, NE, USA) which allows for the simultaneous detection of two proteins in the 700 and 800 channels. Densitometry analysis was performed using Odyssey software (LI-COR, Lincoln, NE, USA). Antibodies for Western blots were as follows: HO-1 (Enzo, Plymouth Meeting, PA, USA), PGC-1α (Milipore, Temecula, CA, USA), Adiponectin, SIRT1, RFP (dsRED), and β-actin (Abcam, Cambridge, MA, USA). All antibodies were used at a ratio of 1:1000 with blocking buffer, the lone exception being β-actin which was used at a ratio of 1:5000. All blots from tissue samples were run with at least 3 samples from all groups of mice per gel.

### 4.7. Statistics

All data are presented as mean ± S.E.M. Differences between treatment groups were determined using one-way analysis of variance with a post hoc test (Dunnett’s). A *p* < 0.05 was considered to be significant. All analyses were performed with SigmaStat (Systat Software, Inc., Richmond, CA, USA).

## 5. Conclusions

In conclusion, we utilized a novel model of adipose-specific knockout of HO-1 to determine its role in the regulation of body weight, composition and fasting blood glucose and insulin levels. We crossed Flox HO-1 mice with mice expressing the Cre recombinase under the control of the adiponectin promoter to achieve adipocyte-specific deletion of HO-1. Adipocyte-specific loss of HO-1 had no significant effect on body weight, composition, fasting glucose or insulin levels in male mice fed either a normal or high-fat diet for 30 weeks. In contrast, adipocyte-specific knockout of HO-1 resulted in increased fat mass, fasting hyperglycemia, and insulinemia in female mice fed both high and normal fat diets. These results suggest that adipocyte HO-1 plays a greater protective role in females versus males and strategies to preserve adipocyte HO-1 may have greater overall metabolic effects in females than in males.

## Figures and Tables

**Table 1:** Body weight and fasting blood glucose in Flox and KO mice fed a normal fat diet for 30 weeks. N.S. = not statistically significant.

Parameter	Sex	Flox	KO	*p*
Body Weight (grams)	Males	31 ± 1 (*n* = 9)	31 ± 0.5 (*n* = 11)	N.S.
Body Weight (grams)	Females	22 ± 0.6 (*n* = 6)	25 ± 0.6 (*n* = 6)	0.03
Fasting blood glucose (mg/dL)	Males	114 ± 5 (*n* = 9)	118 ± 5 (*n* = 11)	N.S.
Fasting blood insulin (ng/mL)	Males	0.7 ± 0.12 (*n* = 6)	0.91 ± 0.29 (*n* = 6)	N.S.
Fasting blood glucose (mg/dL)	Females	99 ± 5 (*n* = 6)	119 ± 5 (*n* = 6)	0.01
Fasting blood insulin (ng/mL)	Females	0.59 ± 0.03 (*n* = 6)	0.82 ± 0.05 (*n* = 6)	0.007

## References

[1] Collaboration N.C.D.R.F. (2016). Trends in adult body-mass index in 200 countries from 1975 to 2014: A pooled analysis of 1698 population-based measurement studies with 19.2 million participants. Lancet.

[2] Mathieu P., Lemieux I., Despres J.P. (2010). Obesity, inflammation, and cardiovascular risk. Clin. Pharmacol. Ther..

[3] Lazo M., Hernaez R., Eberhardt M.S., Bonekamp S., Kamel I., Guallar E., Koteish A., Brancati F.L., Clark J.M. (2013). Prevalence of nonalcoholic fatty liver disease in the united states: The third national health and nutrition examination survey, 1988–1994. Am. J. Epidemiol..

[4] Masuoka H.C., Chalasani N. (2013). Nonalcoholic fatty liver disease: An emerging threat to obese and diabetic individuals. Ann. N. Y. Acad. Sci..

[5] Abraham N.G., Kappas A. (2008). Pharmacological and clinical aspects of heme oxygenase. Pharmacol. Rev..

[6] Peterson S.J., Drummond G., Kim D.H., Li M., Kruger A.L., Ikehara S., Abraham N.G. (2008). L-4f treatment reduces adiposity, increases adiponectin levels, and improves insulin sensitivity in obese mice. J. Lipid Res..

[7] Li M., Peterson S., Husney D., Inaba M., Guo K., Kappas A., Ikehara S., Abraham N.G. (2007). Long-lasting expression of HO-1 delays progression of type I diabetes in nod mice. Cell Cycle.

[8] Li M., Kim D.H., Tsenovoy P.L., Peterson S.J., Rezzani R., Rodella L.F., Aronow W.S., Ikehara S., Abraham N.G. (2008). Treatment of obese diabetic mice with a heme oxygenase inducer reduces visceral and subcutaneous adiposity, increases adiponectin levels, and improves insulin sensitivity and glucose tolerance. Diabetes.

[9] Burgess A., Li M., Vanella L., Kim D.H., Rezzani R., Rodella L., Sodhi K., Canestraro M., Martasek P., Peterson S.J. (2010). Adipocyte heme oxygenase-1 induction attenuates metabolic syndrome in both male and female obese mice. Hypertension.

[10] Ndisang J.F., Jadhav A. (2009). Up-regulating the hemeoxygenase system enhances insulin sensitivity and improves glucose metabolism in insulin-resistant diabetes in goto-kakizaki rats. Endocrinology.

[11] Ndisang J.F., Lane N., Syed N., Jadhav A. (2010). Up-regulating the heme oxygenase system with hemin improves insulin sensitivity and glucose metabolism in adult spontaneously hypertensive rats. Endocrinology.

[12] Csongradi E., Docarmo J.M., Dubinion J.H., Vera T., Stec D.E. (2012). Chronic HO-1 induction with cobalt protoporphyrin (copp) treatment increases oxygen consumption, activity, heat production and lowers body weight in obese melanocortin-4 receptor-deficient mice. Int. J. Obes..

[13] Huang J.Y., Chiang M.T., Chau L.Y. (2013). Adipose overexpression of heme oxygenase-1 does not protect against high fat diet-induced insulin resistance in mice. PLoS ONE.

[14] Ndisang J.F., Lane N., Jadhav A. (2009). The heme oxygenase system abates hyperglycemia in zucker diabetic fatty rats by potentiating insulin-sensitizing pathways. Endocrinology.

[15] Vanella L., Li Volti G., Guccione S., Rappazzo G., Salvo E., Pappalardo M., Forte S., Schwartzman M.L., Abraham N.G. (2013). Heme oxygenase-2/adiponectin protein-protein interaction in metabolic syndrome. Biochem. Biophys. Res. Commun..

[16] Berg A.H., Scherer P.E. (2005). Adipose tissue, inflammation, and cardiovascular disease. Circ. Res..

[17] Oh D.K., Ciaraldi T., Henry R.R. (2007). Adiponectin in health and disease. Diabetes Obes. Metab..

[18] Shibata R., Ouchi N., Murohara T. (2009). Adiponectin and cardiovascular disease. Circ. J..

[19] Kruger A.L., Peterson S., Turkseven S., Kaminski P.M., Zhang F.F., Quan S., Wolin M.S., Abraham N.G. (2005). D-4f induces heme oxygenase-1 and extracellular superoxide dismutase, decreases endothelial cell sloughing, and improves vascular reactivity in rat model of diabetes. Circulation.

[20] Issan Y., Hochhauser E., Kornowski R., Leshem-Lev D., Lev E., Sharoni R., Vanella L., Puri N., Laniado-Schwartzman M., Abraham N.G. (2012). Endothelial progenitor cell function inversely correlates with long-term glucose control in diabetic patients: Association with the attenuation of the heme oxygenase-adiponectin axis. Can. J. Cardiol..

[21] Eguchi J., Wang X., Yu S., Kershaw E.E., Chiu P.C., Dushay J., Estall J.L., Klein U., Maratos-Flier E., Rosen E.D. (2011). Transcriptional control of adipose lipid handling by IRF4. Cell Metab..

[22] Mamiya T., Katsuoka F., Hirayama A., Nakajima O., Kobayashi A., Maher J.M., Matsui H., Hyodo I., Yamamoto M., Hosoya T. (2008). Hepatocyte-specific deletion of heme oxygenase-1 disrupts redox homeostasis in basal and oxidative environments. Tohoku J. Exp. Med..

[23] Hoyenga K.B., Hoyenga K.T. (1982). Gender and energy balance: Sex differences in adaptations for feast and famine. Physiol. Behav..

[24] Shi H., Clegg D.J. (2009). Sex differences in the regulation of body weight. Physiol. Behav..

[25] Lovejoy J.C., Sainsbury A., The Stock Conference 2008 Working Group (2009). Sex differences in obesity and the regulation of energy homeostasis. Obes. Rev..

[26] Reckelhoff J.F., Roman R.J. (2011). Androgens and hypertension: Role in both males and females?. Hypertension.

[27] Maranon R., Reckelhoff J.F. (2013). Sex and gender differences in control of blood pressure. Clin. Sci..

[28] Toth B., Yokoyama Y., Kuebler J.F., Schwacha M.G., Rue L.W., Bland K.I., Chaudry I.H. (2003). Sex differences in hepatic heme oxygenase expression and activity following trauma and hemorrhagic shock. Arch. Surg..

[29] Bonacasa B., Perez C., Salom M.G., Lopez B., Saez-Belmonte F., Martinez P., Casas T., Fenoy F.J., Rodriguez F. (2013). Sexual dimorphism in renal heme-heme oxygenase system in the streptozotocin diabetic rats. Curr. Pharm. Des..

[30] Posa A., Kupai K., Menesi R., Szalai Z., Szabo R., Pinter Z., Palfi G., Gyongyosi M., Berko A., Pavo I. (2013). Sexual dimorphism of cardiovascular ischemia susceptibility is mediated by heme oxygenase. Oxid. Med. Cell. Longev..

[31] Manneras-Holm L., Benrick A., Stener-Victorin E. (2014). Gene expression in subcutaneous adipose tissue differs in women with polycystic ovary syndrome and controls matched pair-wise for age, body weight, and body mass index. Adipocyte.

[32] Kim D.H., Burgess A.P., Li M., Tsenovoy P.L., Addabbo F., McClung J.A., Puri N., Abraham N.G. (2008). Heme oxygenase-mediated increases in adiponectin decrease fat content and inflammatory cytokines tumor necrosis factor-α and interleukin-6 in zucker rats and reduce adipogenesis in human mesenchymal stem cells. J. Pharmacol. Exp. Ther..

[33] Ndisang J.F. (2015). Role of the heme oxygenase-adiponectin-atrial natriuretic peptide axis in renal function. Curr. Pharm. Des..

[34] Yang M., Kimura M., Ng C., He J., Keshvari S., Rose F.J., Barclay J.L., Whitehead J.P. (2015). Induction of heme-oxygenase-1 (HO-1) does not enhance adiponectin production in human adipocytes: Evidence against a direct HO-1-adiponectin axis. Mol. Cell. Endocrinol..

[35] Metoyer C.F., Pruitt K. (2008). The role of sirtuin proteins in obesity. Pathophysiology.

[36] Nogueiras R., Habegger K.M., Chaudhary N., Finan B., Banks A.S., Dietrich M.O., Horvath T.L., Sinclair D.A., Pfluger P.T., Tschop M.H. (2012). Sirtuin 1 and sirtuin 3: Physiological modulators of metabolism. Physiol. Rev..

[37] Liu X., Gao Y., Li M., Geng C., Xu H., Yang Y., Guo Y., Jiao T., Fang F., Chang Y. (2015). Sirt1 mediates the effect of the heme oxygenase inducer, cobalt protoporphyrin, on ameliorating liver metabolic damage caused by a high-fat diet. J. Hepatol..

[38] Sodhi K., Puri N., Favero G., Stevens S., Meadows C., Abraham N.G., Rezzani R., Ansinelli H., Lebovics E., Shapiro J.I. (2015). Fructose mediated non-alcoholic fatty liver is attenuated by HO-1-SIRT1 module in murine hepatocytes and mice fed a high fructose diet. PLoS ONE.

[39] Willis D., Moore A.R., Frederick R., Willoughby D.A. (1996). Heme oxygenase: A novel target for the modulation of the inflammatory response. Nat. Med..

[40] Kushida T., Li V.G., Quan S., Goodman A., Abraham N.G. (2002). Role of human heme oxygenase-1 in attenuating TNF-α-mediated inflammation injury in endothelial cells. J. Cell. Biochem..

[41] Sawle P., Foresti R., Mann B.E., Johnson T.R., Green C.J., Motterlini R. (2005). Carbon monoxide-releasing molecules (co-RMS) attenuate the inflammatory response elicited by lipopolysaccharide in raw264.7 murine macrophages. Br. J. Pharmacol..

[42] Vogel M.E., Zucker S.D. (2016). Bilirubin acts as an endogenous regulator of inflammation by disrupting adhesion molecule-mediated leukocyte migration. Inflamm. Cell Signal..

[43] Hosick P.A., AlAmodi A.A., Storm M.V., Gousset M.U., Pruett B.E., Gray W., Stout J., Stec D.E. (2014). Chronic carbon monoxide treatment attenuates development of obesity and remodels adipocytes in mice fed a high-fat diet. Int. J. Obes. (Lond).

[44] Vera T., Kelsen S., Yanes L.L., Reckelhoff J.F., Stec D.E. (2007). HO-1 induction lowers blood pressure and superoxide production in the renal medulla of angiotensin ii hypertensive mice. Am. J. Physiol. Regul. Integr. Comp. Physiol..

[45] Vera T., Kelsen S., Stec D.E. (2008). Kidney-specific induction of heme oxygenase-1 prevents angiotensin II hypertension. Hypertension.

[46] Csongradi E., Storm M.V., Stec D.E. (2012). Renal inhibition of heme oxygenase-1 increases blood pressure in angiotensin II-dependent hypertension. Int. J. Hypertens..

